# COVID-19 and the ethnicity link – is there a photochemical link?

**DOI:** 10.1007/s43630-020-00004-8

**Published:** 2021-01-08

**Authors:** Ruth Edge, T. George Truscott

**Affiliations:** 1 Dalton Cumbrian Facility, Westlakes Science Park, The University of Manchester, Cumbria, CA24 3HA UK; 2 School of Chemical and Physical Sciences, Keele University, Staffordshire, ST5 5BG UK

**Keywords:** Coronavirus, COVID-19, Sars-CoV-2, Phaeomelanin, Free radicals

## Abstract

A hypothesis is proposed to explain the increased detrimental effect of COVID-19 for Black, Asian and Minority Ethnic (BAME) men and women compared to Caucasian individuals. This is based on the differing photochemistry of phaeomelanin in fair skin and eumelanin in dark/black skin. It is suggested that a range of reactive oxygen species, including, singlet oxygen and the superoxide radical anion, derived via direct photolysis of phaeomelanin, may escape the melanocyte and cause subsequent damage to the SARS-CoV-2 virus. It is further suggested that (large) carbon and sulphur peroxy radicals, from oxygen addition to radicals formed by carbon–sulphur bond cleavage, may assist via damage to the cell membranes. It is also speculated that light absorption by phaeomelanin and the subsequent C-S bond cleavage, leads to release of pre-absorbed reactive oxygen species, such as singlet oxygen and free radicals, which may also contribute to an enhanced protective effect for fair-skinned people.

It is now well established, but not yet understood, that COVID-19 is more damaging, for Black, Asian and Minority Ethnic (BAME) men and women and, even allowing for social aspects there still appears to be an increased detrimental effect, with Black Africans having the highest death rates (by a factor of two compared with White (Caucasian) individuals) [1, 2]. Of course, BAME people, particularly Black Africans, have much darker skin types, e.g., skin types IV-V (brown to black) or type VI (very black) which contain an increased amount of melanin pigment. BAME individuals could be both at increased risk of acquiring the disease as well as an increased disease severity, susceptibility or response to infection.

Since no molecular mechanism is established for the increased risk of COVID-19 among BAME communities, it is worth considering possible photochemical processes in the skin, generating and releasing activated oxygen species, free radicals, metal ions.

One possible reason for an increased susceptibility that has been discussed is their higher prevalence of Vitamin D deficiency. While there is strong support for the beneficial value of vitamin D [3–5], there are reports that do not support a potential link between vitamin D concentrations and risk of COVID-19 infection, nor that vitamin D concentration may explain ethnic differences in COVID-19 infection [6]. If vitamin D is relevant there is, of course, an indirect photochemical link associated with vitamin D synthesis and light transmission through the skin. Of course, all photochemical processes will be related to light exposure, i.e., to seasonal and social factors.

Skin colour and photo-protection are related to melanin type (Eumelanin [EM] and Phaeomelanin [PM]) and their levels/concentrations/size of pigment granules. Melanogenesis of both types starts with the generation of dopaquinone, the initial product of tyrosine oxidation by tyrosinase, with a detailed kinetic understanding now established [7]. This process then diverges and, for eumelanogenis, cyclodopa is formed which proceeds rapidly to produce dopachrome while for phaeomelanogenis cysteine addition leads to cysteinyldopa and then to benzothiazoles—these are a characteristic of PM [7]. EM and PM can exist as intimate mixtures in epidermal areas of skin pigmentation [8]. EM protects skin and increases with light exposure while PM causes the susceptibility of fair-skinned individuals to the deleterious effects of sunlight. In addition, of course, there is deeper penetration of light in such fair skin compared to dark/black skin [9].

Two aspects of specific ROS component generation must be considered; (i) the direct generation of ROS such as free radicals, metal ions and singlet oxygen and (ii) an indirect route via melanin structural changes resulting in the release of such damaging species which have been incapsulated by the melanins as they are formed.

Direct ROS generation: as noted above phaeomelanogenesis is responsible for the increased susceptibility of fair-skinned phaeomelonic individuals to the deleterious effects of sunlight and this is due to the photo-processes following light absorption by phaeomelanin [10]. Photochemical studies of models of PM show that this melanin can produce singlet oxygen, the superoxide radical anion (O_2_^•−^) and carbon and sulphur-based radicals [10–12]. Also melanins chelate metals especially iron [13, 14] leading to Fenton chemistry, which is a route to producing hydroxyl radicals from superoxide.

For PM, the damaging radical superoxide (O_2_^•−^) and, less likely, see below, activated (singlet) oxygen could be particularly important because they may be able to undergo trans-membrane processes—discussed later. Of course, cells are protected from O_2_^•−^ by superoxide dismutase (SOD) [15].

Our early work, over 30 years ago [11], on PM models, showed unambiguously that light causes the production of the melanin triplet states (via sensitisation of the β-carotene triplet). Such triplets will, of course, undergo energy transfer with oxygen to give the damaging ROS known as singlet oxygen. Others have also shown SO generation and, importantly, that melanins quench such species, with EM being a more efficient quencher than PM [16, 17]. So, for the fair-skinned, there may be a ‘compounded’ effect with the yield of singlet oxygen generated being higher for PM than EM and also less quenching of the singlet oxygen by the reduced EM concentration. This aspect has been comprehensively reviewed by Ito and co-workers who also discuss the generation of O_2_^•−^ and the light induced structural modifications of both melanins [18].

An important aspect of PM photo-destruction is cleavage of the C-S bonds leading to the corresponding S-based and C-based neutral free radicals [12]. We have recently shown, in human cells/lymphocytes, neutral free radicals can add oxygen to generate the corresponding peroxyl radical and this species can be a particularly effective at cell membrane damage [15, 19]. The effectiveness depending on the position of the equilibrium for the systems studied.

$$ {\text{R}}^{ \bullet }  + {\text{O}}_{2}  \rightleftarrows {\text{RO}}_{2} ^{ \bullet } $$

A possible role of such peroxyl species relating to membrane damage is discussed later.

Indirect ROS generation: Here we are not concerned with generating such species directly with light but with releasing ROS due to the photo-modification of the melanins, these ROS having originally been accumulated internally into the melanin granule by the melanogenis processes itself. It is well established that free radicals within the melanins can be detected via ESR [20, 21] and this is increased by UVA and B, visible light and even red/infra-red ‘light’ [22]. In this study skin types IV–V were used, with the darker skin types based on volunteers of African, South American and Indian origin, and showed increased radical formation from near infra-red irradiation [22]. Clearly, such low energy radiation does not generate ‘new’ free radicals via bond cleavage. The reasons for this observation are not clear but may involve the radiation causing bond disruption and possibly bond rotations in the melanin macromolecule with this leading to structural changes allowing the pre-absorbed radicals to escape. Whatever, of course, EM is photoprotective against radiation and the deleterious effects are due to PM. However, it should be noted that the PM and EM in vivo are not polymers which lie discreetly ‘side by side’. By analogy to neuromelanin [23], they are arranged in melanosomes so that a core of PM is surrounded by EM polymers, with the thickness of the eumelanic exterior diminishing as the ratio of PM to EM increases. So, we also propose the change of shape of PM due to photocleavage and other structural rearrangements are processes which cause, as a secondary effect, the EM to rearrange and release some of its bound free radicals, without necessarily involving direct light absorption by EM. Indeed, as noted above, EM may quench some of the ROS produced by PM, such as singlet oxygen.

Two critical aspects must be considered for our hypothesis to be feasible and worthy of detailed consideration. Firstly, can any PM photolysis products escape from melanocytes and, secondly, are their lifetimes sufficiently long to interact with other species in the blood stream, especially the virus particle. We consider both these questions below.

Dealing first with possible escape processes:

As noted above [18] a complex range of structural changes of both EM and PM can arise by the absorption of specific wavelengths of light. We suggest, for PM, another possible mechanism is that (large) peroxyl radicals such as RSO_2_^•^ assist the escape of smaller ROS, such as singlet oxygen and O_2_^•−^ from the melanin particles via membrane damage. Furthermore, it has been shown that singlet oxygen can cross model cell membranes [24]. For O_2_^•−^ it is generally thought [25] such a trans-membrane route is inefficient because the O_2_^•−^ is charged and any escape would need to be via H_2_O_2._ However, a review of redox signalling across cell membranes has shown, in addition to the route via H_2_O_2_, a direct chloride channel can allow direct membrane crossing [26]. Another possibility is that certain membrane-spanning molecules, such as astaxanthin, can act as radical transfer bridges [27]. Strong evidence for the escape comes from the ex-vivo ESR studies of human skin by Zastrow and co-workers [28]. Taking into account the depth of penetration of sunlight these workers showed the radicals produced can interact with blood or lymphatic liquids so that systemic action can be expected.

Of course, from early work, as discussed above [10], this seems feasible because O_2_^•−^ can be detected by a colourmetric measurement with nitroblue tetrazolium following UV/visible light exposure of PM. However, it is also well established that PM (and EM) react with free radicals [29]. So, a confusing aspect of PM photochemistry is that it not only generates a wide range of ROS it also reacts with these same species – i.e., it is both an initiator and victim of oxidative stress! Of course, despite such reactions, PM still leads to significant photosensitivity so its reactions with ROS do not prevent the generation and escape of some ROS such as O_2_^•−^. Indeed, as shown previously [10] if there is a direct reaction of PM with O_2_^•−^ it is not fast enough to prevent O_2_^•−^ escaping to the free solution.

Now dealing with lifetimes:

## Singlet oxygen

The role of SO is worthy of discussion even though it may be too short lived to be of relevance to this hypothesis. Studies using D_2_O (lifetime of SO = 56 μs) [24] show it is sufficiently long-lived to migrate through the ‘aqueous’ solution and cross into a DPPC membrane before reacting with lycopene and β-Carotene which are embedded in the membrane far from the aqueous boundary. Furthermore, the SO quenching reaction rate constant, compared to the situation where the SO is generated in the membrane itself, is identical–that is, there is no measurable decay of the SO while it migrates from its source in water (D_2_O) to the centre of the lipid bilayer. The environment of the melanocyte is heterogeneous but primarily ‘organic’ where the SO lifetime can range from about 10 μs in ethanol to 250 μs in chloroform and 26,000 μs in CCl_4_ [30], so it is certainly feasible that the SO has time to escape from the melanocyte into the blood stream. Of course, once in the blood stream, the lifetime is much reduced (to 4.2 μs in H_2_O – a factor of 13 times compared to D_2_O) [31] so it may well have significantly decayed (minimum 6% left) before it can contribute to our hypotheses of virus inactivation. Nevertheless, our estimate of SO lifetime being reduced to only 6% of that in an organic environment, is a minimum value. So while a possible role for SO seems unlikely it should not be totally dismissed since we saw zero deactivation of SO in D_2_O for migration into the lipid bilayer [24]. Another aspect is that EM quenches SO itself, but, of course, there is reduced EM in fair skinned people so this route to deactivation of SO is less significant.

## Superoxide radical anion

Here the situation is quite different to SO. Generally, it is known as a relatively unreactive species. In organic solvents O_2_^•−^ is stable, i.e., has an extremely long lifetime, while in water it is much shorter lived due to a range of possible reactions [32]. As noted for SO the O_2_^•−^ will be generated from light absorption by PM in a mainly organic environment so will have time to enter the bloodstream provided it can escape from the PM. Basically, there are two initial consequences of O_2_^•−^. It can be rendered harmless, as presumably happens with SOD or it can react to generate another damaging species such as with nitric oxide to give the non-radical but damaging peroxynitrite (O_2_^•−^  + NO → OONO^−^).

One important finding, as noted above, is that a very recent study [33] shows the coronavirus affects endothelial cells in the blood vessels. So, the radicals which escape the melanocytes may not need to travel to the lungs and respiratory tract but just need to reach the blood and thus their lifetime may be adequate for reaction with/deactivation of the virus.

Furthermore, the fact that viruses such as COVID-19 does not contain SOD may be important in this respect.

The authors of the research on COVID-19 endothelial cell injury [33] also state ‘early identification of endotheliopathy and strategies to mitigate its progression might improve outcomes in COVID-19′. Given the article of Zastrow et al*.* [28] on human skin indicating photolytically generated radicals can reach the blood and the fact that blood irradiation was used in the 1940s and 50 s to treat patients with pneumonia, TB, sepsis and asthma (among other diseases) [34], it seems feasible to hypothesise that phototherapy could ease endotheliopathy due to COVID-19 and, in general, that those with paler skins may need lower levels of light to generate beneficial effects.

Additionally, very old work by Knott [35] suggested that at least some of the beneficial effects of UV skin irradiation could be due to direct irradiation of the blood circulating in the skin’s capillaries. Whilst this effect may not be directly related to any radical production from melanin, the amount of light penetrating paler skins is higher [9] meaning more will be able to reach the bloodstream.

## C- and S-based radicals

PM, like EM is a huge polymeric molecule with many thousands of monomer units. While little is known about the molecular processes following light and near UV absorption [18], it is destroyed. Breakage, and subsequent cross linking, of just one C–C or C-S bond can itself lead to many thousands of products depending on where the photolysis occurs. Subsequent photolysis, over and over, would end up with a substantial number of the molecular units which are the precursors of the PM such as 5-S-cysteinyldopa (HO)_2_RC_6_H_2_SCH_2_CH(COO-)NH_3_^+^. It was established over 40 years ago that the concentration of this species in the serum increases by 100–300% in fair skinned humans following exposure to sunlight [36]. The primary photochemical events in 5-SCD photolysis generates sulphur [(HO)_2_RC_6_H_2_S^•^] radicals and carbon [(^•^CH_2_CH(COO −)NH_3_^+^] radicals. Both these are likely to form reactive peroxyl radicals via molecular oxygen addition. The sulphur containing thiyl and thiyl-sulphonyl radicals can readily initiate lipid peroxidation and can also form long-lived, resonance stabilised adducts with organic bio-substrates species [37]. We suggest such reactive sulphur based radicals may damage lipid bilayers assisting the escape of smaller reactive species such as O_2_^•−^ and possibly species generated via iron catalysed Fenton chemistry, to escape into the blood stream.

In this hypothesis we are not concerned with comparing the detailed mechanism of specific ROS with EM and PM [18] nor with mechanisms which may lead to the destruction of the COVID-19 virus. Rather, we have suggested virus-damaging ROS can arise from skin melanins via several distinct routes. Furthermore, it may be noted (i) there are claims that smoking, which leads to the production of free radicals, may reduce COVID-19 infection risk [38] and (ii) the COVID-19 spikes are rich in cysteine [39–41] and this amino acid is readily destroyed by ROS, such as singlet oxygen and O_2_^•−^.

Overall, taking a simplistic view, it may appear counter-intuitive that skin photo-protection and reduced damaging pro-oxidative species from eumelanin photochemistry may be linked to the increased severity of COVID-19 in dark skinned BAME. However, whatever the precise molecular mechanism leading to partial protection from the COVID-19 virus for fair-skinned individuals, we propose this may arise from the reactive species generated from PM photolysis and rearrangement, with deeper light penetration for people with skin types I-III (fair-skinned), in contrast to low light penetration in people with skin types IV–V (dark-skinned) and virtually none at all in those with Type VI black skin. We note^1^ that native Americans have high PM content in their skin and from CDC data (Centers for Disease Control and Prevention), although the number of coronavirus cases per 100,000 in American Indian or Alaskan natives is 2.8 times higher than for the US white population, the death rate per 100,000 is only 1.4 × higher. This is compared to a 2.6 times higher case rate for Black and African Americans with a 2.1 times higher death rate [42].

In conclusion, we suggest that photochemistry should be considered as a mechanism for the ethnicity links to COVID-19.

## Data Availability

Not applicable.

## Footnotes

1 We thank referee 3 for informing us of this point.
